# The BBX gene *CmBBX22* negatively regulates drought stress tolerance in chrysanthemum

**DOI:** 10.1093/hr/uhac181

**Published:** 2022-08-25

**Authors:** Yanan Liu, Hua Cheng, Peilei Cheng, Chunmeng Wang, Jiayu Li, Ye Liu, Aiping Song, Sumei Chen, Fadi Chen, Likai Wang, Jiafu Jiang

**Affiliations:** State Key Laboratory of Crop Genetics and Germplasm Enhancement, Key Laboratory of Landscaping, Ministry of Agriculture and Rural Affairs, Key Laboratory of Biology of Ornamental Plants in East China, National Forestry and Grassland Administration, College of Horticulture, Nanjing Agricultural University, Nanjing 210095, China; State Key Laboratory of Crop Genetics and Germplasm Enhancement, Key Laboratory of Landscaping, Ministry of Agriculture and Rural Affairs, Key Laboratory of Biology of Ornamental Plants in East China, National Forestry and Grassland Administration, College of Horticulture, Nanjing Agricultural University, Nanjing 210095, China; State Key Laboratory of Crop Genetics and Germplasm Enhancement, Key Laboratory of Landscaping, Ministry of Agriculture and Rural Affairs, Key Laboratory of Biology of Ornamental Plants in East China, National Forestry and Grassland Administration, College of Horticulture, Nanjing Agricultural University, Nanjing 210095, China; State Key Laboratory of Crop Genetics and Germplasm Enhancement, Key Laboratory of Landscaping, Ministry of Agriculture and Rural Affairs, Key Laboratory of Biology of Ornamental Plants in East China, National Forestry and Grassland Administration, College of Horticulture, Nanjing Agricultural University, Nanjing 210095, China; State Key Laboratory of Crop Genetics and Germplasm Enhancement, Key Laboratory of Landscaping, Ministry of Agriculture and Rural Affairs, Key Laboratory of Biology of Ornamental Plants in East China, National Forestry and Grassland Administration, College of Horticulture, Nanjing Agricultural University, Nanjing 210095, China; State Key Laboratory of Crop Genetics and Germplasm Enhancement, Key Laboratory of Landscaping, Ministry of Agriculture and Rural Affairs, Key Laboratory of Biology of Ornamental Plants in East China, National Forestry and Grassland Administration, College of Horticulture, Nanjing Agricultural University, Nanjing 210095, China; State Key Laboratory of Crop Genetics and Germplasm Enhancement, Key Laboratory of Landscaping, Ministry of Agriculture and Rural Affairs, Key Laboratory of Biology of Ornamental Plants in East China, National Forestry and Grassland Administration, College of Horticulture, Nanjing Agricultural University, Nanjing 210095, China; State Key Laboratory of Crop Genetics and Germplasm Enhancement, Key Laboratory of Landscaping, Ministry of Agriculture and Rural Affairs, Key Laboratory of Biology of Ornamental Plants in East China, National Forestry and Grassland Administration, College of Horticulture, Nanjing Agricultural University, Nanjing 210095, China; State Key Laboratory of Crop Genetics and Germplasm Enhancement, Key Laboratory of Landscaping, Ministry of Agriculture and Rural Affairs, Key Laboratory of Biology of Ornamental Plants in East China, National Forestry and Grassland Administration, College of Horticulture, Nanjing Agricultural University, Nanjing 210095, China; State Key Laboratory of Crop Genetics and Germplasm Enhancement, Key Laboratory of Landscaping, Ministry of Agriculture and Rural Affairs, Key Laboratory of Biology of Ornamental Plants in East China, National Forestry and Grassland Administration, College of Horticulture, Nanjing Agricultural University, Nanjing 210095, China; State Key Laboratory of Crop Genetics and Germplasm Enhancement, Key Laboratory of Landscaping, Ministry of Agriculture and Rural Affairs, Key Laboratory of Biology of Ornamental Plants in East China, National Forestry and Grassland Administration, College of Horticulture, Nanjing Agricultural University, Nanjing 210095, China

## Abstract

BBX transcription factors play vital roles in plant growth, development, and stress responses. Although BBX proteins have been studied in great detail in the model plant *Arabidopsis*, their roles in crop plants such as chrysanthemum are still largely uninvestigated. Here, we cloned *CmBBX22* and further determined the function of *CmBBX22* in response to drought treatment. Subcellular localization and transactivation assay analyses revealed that CmBBX22 was localized in the nucleus and possessed transactivation activity. Overexpression of *CmBBX22* in chrysanthemum was found to reduce plant drought tolerance, whereas expression of the chimeric repressor *CmBBX22-SRDX* was found to promote a higher drought tolerance than that shown by wild-type plants, indicating that *CmBBX22* negatively regulates drought tolerance in chrysanthemum. Transcriptome analysis and physiological measurements indicated the potential involvement of the *CmBBX22*-mediated ABA response, stomatal conductance, and antioxidant responses in the negative regulation of drought tolerance in chrysanthemum. Based on the findings of this study, we were thus able to establish the mechanisms whereby the transcriptional activator CmBBX22 negatively regulates drought tolerance in chrysanthemum via the regulation of the abscisic acid response, stomatal conductance, and antioxidant responses.

## Introduction

Plants are adversely affected by a diverse range of biotic and abiotic stress factors, among which drought stress is potentially one of the most serious adverse factors disrupting plant metabolism, photosynthesis, and cell structure, thereby ultimately reducing plant growth [1, 2]. Consequently, it is of particular importance to study the response of plants to drought stress, thereby providing a theoretical basis for enhancing the drought tolerance of plants [3].

Zinc finger-containing proteins encode a family of plant-specific transcription factors that play significant roles in plant growth and development as well as response to environmental stimuli [4, 5]. The zinc finger family is one of the largest transcription factor families in plants [6, 7]. The BBX proteins are a subgroup of zinc finger transcription factors, carrying one or two conserved B-box domains in their N-terminus, and some members may also have a CCT (CONSTANS, CO-like, and TOC1) domain or a valine-proline (VP) motif in the C terminus [6, 8]. In *Arabidopsis*, it has been reported that the AtBBX family comprises 32 members, which can be divided into five subgroups based on their structural domains [8]. Among these, *CONSTANS* (*CO*/*BBX1*) has been identified as one of the most important factors involved in the regulation of flowering. Overexpression of *CONSTANS* in *Arabidopsis* has been demonstrated to result in earlier flowering than in wild-type (WT) plants under long-day conditions, whereas the findings of several studies have indicated that the BBX proteins play roles in the responses of plants to abiotic stress [9, 10]. In rice, the expression of *OsBBX1*, *OsBBX2*, *OsBBX8*, *OsBBX19*, and *OsBBX24* has been observed to be strongly induced by abiotic stresses such as drought, cold, and salt stresses [11], and the overexpression of *AtBBX21* in potato (*Solanum tuberosum*) plants has been found to promote a higher tolerance to water restriction, along with higher levels of chlorophylls and tuber yield than in WT plants [12]. Similarly, overexpression of the *AtBBX24* (*STO*) gene in *Arabidopsis* results in a higher salt tolerance [13], whereas knockdown of *CmBBX24* in chrysanthemum has the effect of reducing tolerance to freezing and drought stresses, which has been found to be associated with the downregulation of genes related to carbohydrate metabolism and soluble substances [14]. Furthermore, it has been reported that *AtBBX18* is involved in thermotolerance via regulation of a set of heat-shock-responsive genes [15], whereas recent studies have revealed that CmBBX19 negatively regulates drought tolerance in chrysanthemum [16] and MdBBX7 endows drought tolerance in apple (*Malus domestica*) [17]. Collectively, the findings of these studies provide convincing evidence to indicate that *BBX* genes positively or negatively contribute to the regulation of abiotic stress responses in plant, particularly the response to drought.

Abscisic acid (ABA), one of the plant hormones, regulates multiple important biological processes, including plant development and stress response. Several studies have shown that BBX21 inhibits ABA-regulated seed germination through its binding to the promoter of *ABI5* [18, 19]. In addition, *CmBBX22* improves drought stress resistance in *Arabidopsis* by regulating the ABA signaling pathway [20]. A recent report showed that the BBX19-ABF3 complex plays an important role in modulating drought response in chrysanthemum in an ABA-dependent pathway [16]. Taken together, these studies suggest that some BBX proteins regulate plant development and stress response possibly through ABA signaling.

AtBBX22 contains two B-box domains near its N-terminus. Previous reports showed that AtBBX22 positively regulates photomorphogenesis in *Arabidopsis* through interacting with HY5 and COP1, and its degradation was conducted by COP1-mediated ubiquitination [21, 22]. It has been reported that heterologous expression of *CmBBX22* in *Arabidopsis* leads to delayed leaf senescence and improved drought tolerance [20]. However, the functions of *CmBBX22* in chrysanthemum are still unknown.

Chrysanthemum (*Chrysanthemum morifolium*) is among the most widely cultivated ornamental species worldwide, the productivity and quality of which are materially influenced by environmental stress. In this study, we found that constitutive expression of *CmBBX22* resulted in a heightened sensitivity to drought stress, whereas in contrast constitutively expressing the chimeric repressor *CmBBX22-SRDX* was found to promote a higher level of drought tolerance than that observed in WT plants. Moreover, transcriptome analysis and physiological measurements revealed that *CmBBX22*-mediated stomatal conductance, ABA response, and antioxidant responses are potentially involved in the negative regulation of drought stress in chrysanthemum. Collectively, our findings indicate that *CmBBX22* acts in a negative role in the drought stress response in chrysanthemum.

## Results

### Subcellular localization and transcriptional activity of the CmBBX22 protein

**Figure 1:**
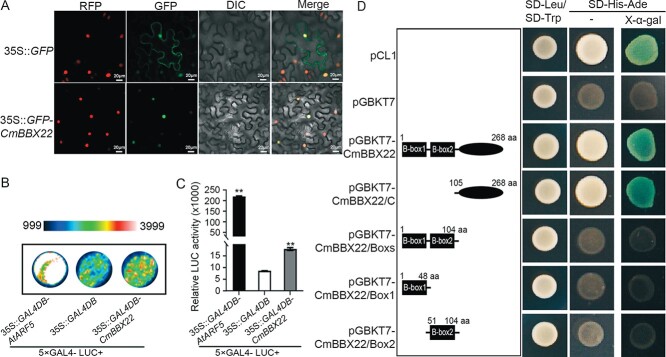
Verification of subcellular localization and transactivation of CmBBX22. (A) Subcellular localization of CmBBX22 in tobacco (*N. benthamiana*) cells. RFP, 35S::D53-RFP construct, which served as the co-localization marker; GFP, pictures taken from the green fluorescence channel; DIC, images captured from bright light; Merged, overlay plots. Bars = 20 μm. (B) Image captured by a low-light cooled CCD. (C) LUC reporter activities of CmBBX22. 5 × GAL4-LUC + 35S::*GAL4DB-AtARF5* served as the positive control; 5 × GAL4-LUC + *35S::GAL4DB* was used as the negative control. Student’s *t*-test was used to analyze significant differences; ^**^P < 0.01. Error bars indicate standard deviation (*n* = 3). (D) Transcriptional activity analysis of CmBBX22 in yeast cells. Left panel: diagram of the five different segments of CmBBX22. pCL1 served as positive control, and pGBKT7 as negative controls. Right panel: results of transactivation activity. SD-Leu, SD medium without leucine; SD-Trp, SD medium without tryptophan; SD-His-Ade, SD medium without histidine and adenine; X-α-gal, SD-His-Ade medium with 20 mM X-α-gal.

CmBBX22 is a member of the B-box (BBX)-containing zinc finger transcription factor family [7, 20]. To investigate the subcellular localization of CmBBX22, we performed transient expression of *CmBBX22* in *Nicotiana benthamiana*, by infiltrating plant leaves with a suspension of *Agrobacterium tumefaciens* carrying the 35S::*GFP-CmBBX22* expression construct or the 35S::*GFP* control vector, together with *35S::D53-RFP* vector as a nuclear localization marker. In plants transformed with the 35S::*GFP* plasmid, green fluorescence signals were detected in both cytoplasm and nucleus (Fig. 1A), whereas, in contrast, fluorescence was detected exclusively in the nuclei of leaf epidermal cells expressing the 35S::*GFP-CmBBX22* plasmid (Fig. 1A), thereby indicating that, similar to other transcription factors, CmBBX22 is a nuclear-localized protein.

To examine the transcriptional activity of CmBBX22, we performed a reporter–effector transient expression assay. Initially, we generated a translational fusion construct, in which the protein-coding region of CmBBX22 was fused to the GAL4 DNA-binding domain (35S::*GAL4DB-CmBBX22*), whereas constructs containing the GAL4 DNA-binding domain linked to the strong activator AtARF5 (35S::*GAL4DB-AtARF5*) were used as a positive control (Supplementary Data Fig. S1) [23]. Intriguingly, we found that, compared with the GAL4-only negative control effector (35S::*GAL4DB*), both the 35S::*GAL4DB-CmBBX22* and 35S::*GAL4DB-AtARF5* effectors promoted an increase in luciferase (LUC) activity in transformed *Arabidopsis* mesophyll protoplasts (Fig. 1B and C), thereby indicating that CmBBX22 has transcriptional activator activity. Further confirmation that CmBBX22 functions as a transcription activator was obtained based on yeast two-hybrid (Y2H) Gold assays (Fig. 1D). Transformation of cells with a construct containing the C-terminal region of the CmBBX22 protein (residues 105–268), pGBKT7-CmBBX22/C, was found to be sufficient to induce activation of the reporter, whereas, in contrast, no comparable expression was detected in cells harboring constructs containing the two Box domains [pGBKT7-CmBBX22-Boxes (residues 1–104)], [pGBKT7-CmBBX22/Box1 (residues 1–48 residues)] or [pGBKT7-CmBBX22/Box2 (residues 51–104)] (Fig. 1D). These findings accordingly indicate that CmBBX22 is a transcription activator and that transcriptional activation is mediated via the C-terminal region of this protein.

### The *CmBBX22* gene negatively regulates drought tolerance in chrysanthemum

**Figure 2:**
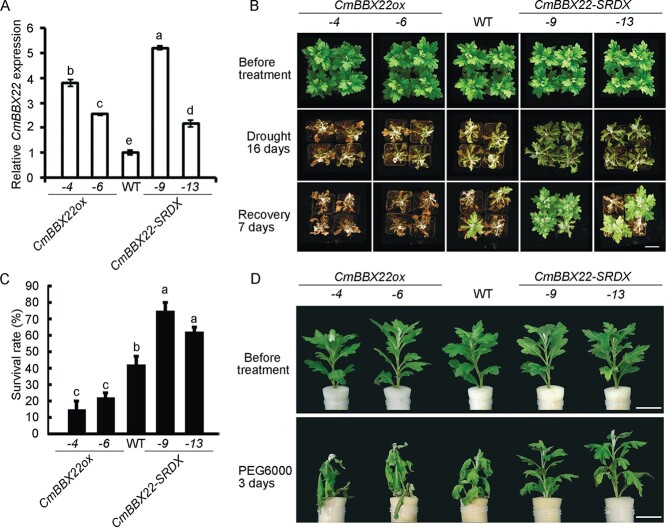
Drought stress response of *CmBBX22*-overexpressing (*CmBBX22ox*) or *CmBBX22*-suppressed (*CmBBX22-SRDX*) chrysanthemum plants. (A) (qRT)–PCR analysis of *CmBBX22* in WT and transgenic lines. *CmBBX22ox-4* and *CmBBX22ox-6* represent two independent *CmBBX22ox* transgenic lines, and *CmBBX22-SRDX-9* and *CmBBX22-SRDX-13* correspond to two independent *CmBBX22-SRDX* lines. Error bars indicate standard deviation (*n* = 3). Significant differences are indicated by different letters (*P* < 0.05), as analyzed by Duncan’s test. (B) Phenotypes of *CmBBX22* transgenic plants and WT plants after water-withholding treatment. Water was withheld from 4-week-old *CmBBX22ox*, *CmBBX22-SRDX*, and WT plants in soil for 16  days (Drought 16 days), and plants recovered for 7 days with regular watering (Recovery 7 days). Three independent assays were performed with similar results. Bar = 5 cm. (C) Survival rates of *CmBBX22ox*, *CmBBX22-SRDX*, and WT plants after 7 days of re-watering following 16-day drought treatment. Three independent experiments were performed; for each experiment (*n* = 9), error bars indicate the standard deviation. Significant differences were determined by Duncan’s test (*P* < 0.05). (D) Phenotypes of *CmBBX22* transgenic plants and WT plants before and after 20% PEG6000 treatment for 3 days. Bar = 3 cm.

To further investigate the function of *CmBBX22* in chrysanthemum, we generated overexpression lines of *CmBBX22* in the cultivar ‘Jinba’ by introducing the plasmid pMDC43-*CmBBX22*, whereas chimeric repressor lines were generated by transforming with the 35S::*CmBBX22-SRDX* construct. Positive transgenic lines were identified by conducting a genomic PCR assay and quantitative real-time PCR (qRT–PCR) analysis (Supplementary Data Fig. S2A; Fig. 2A). We accordingly found that the levels of *CmBBX22* transcription were substantially higher in transgenic lines than in WT plants (Fig. 2A). On the basis of these findings, the *CmBBX22*-overexpressing lines *CmBBX22ox-4* and *CmBBX22ox-6* and the *CmBBX22* chimeric repressor lines *CmBBX22-SRDX-9* and *CmBBX22-SRDX-13* were selected for subsequent analyses (Fig. 2A).

The heterologous expression of *CmBBX22* in *Arabidopsis* has previously been demonstrated to enhance plant drought tolerance [20]. To examine the function of *CmBBX22* in the regulation of drought tolerance in chrysanthemum, we subjected transgenic and WT chrysanthemums to a 16-day period of drought stress. We found that the *CmBBX22ox* lines were severely damaged by drought treatment, whereas *CmBBX22-SRDX* plants exhibited only slight damage compared with WT plants (Fig. 2B). Subsequent to drought treatment, plants were re-watered and continually cultured for a further 7 days, after which time the *CmBBX22ox* plants were found to have significantly lower survival compared with the WT control plants, whereas *CmBBX22-SRDX* plants showed comparatively higher survival (Fig. 2B and C). These observations thus tend to indicate that *CmBBX22* negatively regulates drought stress tolerance in chrysanthemum.

As further confirmation of *CmBBX22* function in the regulation of drought tolerance in chrysanthemum, we subjected transgenic and WT plants to a 20% PEG6000-simulated drought treatment. It was accordingly found that the degree of base-leaf wilting in *CmBBX22ox* plants was more pronounced than that observed in WT plants, whereas no significant impairment was observed in *CmBBX22-SRDX* plants (Fig. 2D), which is consistent with the previous observations that *CmBBX22* negatively regulates drought tolerance in chrysanthemum.

Previous reports showed that the phytohormone ABA regulates drought stress responses and resistance in plants [20, 24]. To examine the effects of ABA on the *CmBBX22*-mediated drought response in chrysanthemum, we performed ABA treatment on detached chrysanthemum leaves. In WT plants, we found that ABA treatment maintained higher leaf water content for a longer period under water deficit conditions than the plants without ABA treatment, possibly due to reduced leaf transpiration by closing stomata (Supplementary Data Fig. S2B). The largest difference between ABA treatment and mock was observed at 6 h (Supplementary Data Fig. S2C). We then performed ABA treatment on the detached leaves from transgenic (*CmBBX22ox-4* and *CmBBX22-SRDX-9*) and WT plants. We found that the *CmBBX22ox* plants had more severely wilted leaves compared with the WT control, whereas *CmBBX22-SRDX* plants wilted slightly (Supplementary Data Fig. S2D), indicating that the *CmBBX22*-mediated ABA response is potentially involved in the negative regulation of chrysanthemum drought tolerance.

### Global expression analysis of genes regulated by *CmBBX22*

**Figure 3:**
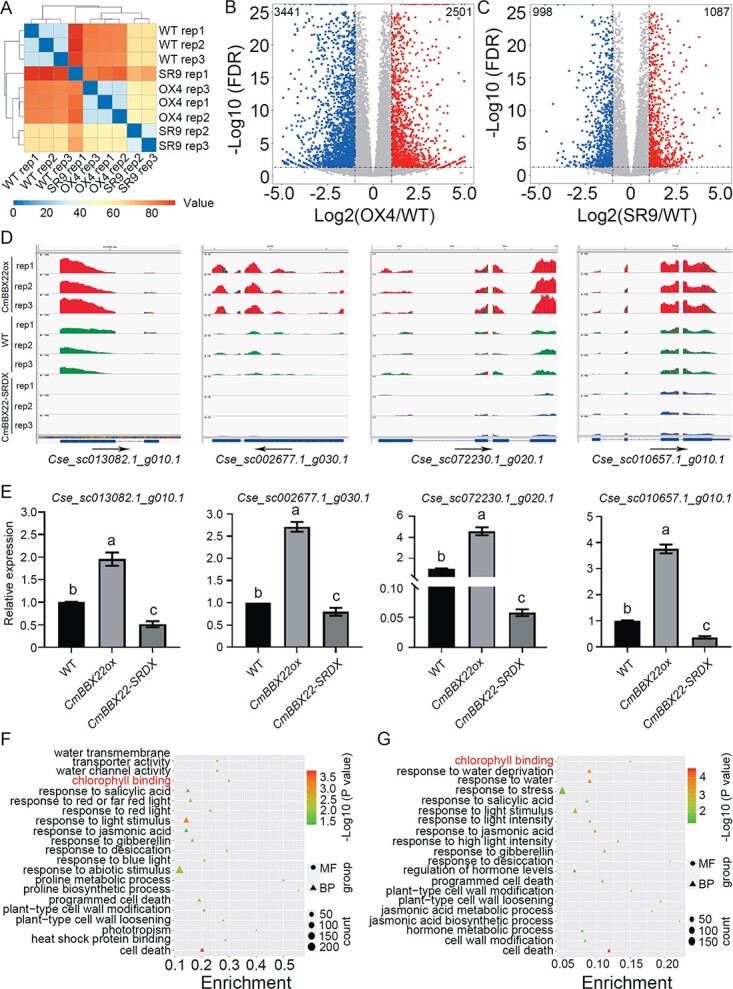
Global expression analysis of genes regulated by *CmBBX22*. (A) Heat map of correlation values for pairwise comparisons between samples. The correlation analysis was performed by DEseq2. OX4 indicates the *CmBBX22ox-4* transgenic plant; SR9 indicates the *CmBBX22-SRDX-9* plant. (B, C) Volcano plots of gene expression changes in *CmBBX22ox* versus WT (B) and *CmBBX22-SRDX* versus WT (C). OX4 indicates the *CmBBX22ox-4* transgenic plant; SR9 indicates the *CmBBX22-SRDX-9* plant. DEGs were selected by *q* < 0.05 and |log2 (fold change)| > 1. The *x*-axis shows the fold change in gene expression between transgenic and WT plant samples, and the *y*-axis shows the statistical significance of the differences. Colors represent different genes: gray for genes without significantly different expression, blue for significantly downregulated genes, and red for significantly upregulated genes. (D) Genome browser traces of RNA-seq results of DEGs in *CmBBX22ox*, *CmBBX22-SRDX*, and WT plants. (E) qRT–PCR assay to examine the expression of genes in *CmBBX22ox*, *CmBBX22-SRDX*, and WT plants. Chrysanthemum *EF1α* was used as the reference gene for normalization. Error bars indicate the standard deviation (*n* = 3). Different letters indicate statistical differences determined by Duncan’s test (*P* < 0.05). (F, G) GO analysis using all the DEGs between *CmBBX22ox* and WT (F) and *CmBBX22-SRDX* and WT (G). RNA-seq analyses were performed using *CmBBX22ox*, *CmBBX22-SRDX*, and WT plants cultivated under normal growth conditions. GO analyses were conducted using 5942 and 2085 DEGs that were identified by comparisons between *CmBBX22ox* and WT and between *CmBBX22-SRDX* and WT plants, respectively. The bubble plot shows enrichment for GO pathways.

To gain more insight into the molecular mechanisms underlying the *CmBBX22-*mediated control of drought tolerance in chrysanthemum, we subsequently performed RNA-seq analysis using *CmBBX22ox*, *CmBBX22-SRDX*, and WT plants cultivated under normal growth conditions, for each type of which three biological replicates were collected for sequencing. Having initially removed adaptor sequences and low-quality reads from the raw sequence data, the remaining clean reads were aligned to the *Chrysanthemum seticuspe* genome release CSE_r1.0 sequence [25] using the kallisto program [26] (Supplementary Data Table S2). Verification of the similarity of three biological replicates of the WT and *CmBBX22ox* plants confirmed that the data were reproducible (Fig. 3A). However, one of the *CmBBX22-SRDX* replicates was found to have large variation compared with the other two replicates (Fig. 3A), and thus we performed subsequent analyses using only replicates 2 and 3. Analysis of the expression of *Cs3_sc004370.1_g0600.1* (*CmBBX22*) obtained by RNA-seq revealed that, in line with the qRT–PCR results (Fig. 2A; Supplementary Data Fig. S3A and B), the transcriptional level of *CmBBX22* in transgenic plants was considerably higher than that in WT plants.

Having characterized the expression of *CmBBX22* in response to drought, we proceeded to identify differentially expressed genes (DEGs) using DEseq2 methods [27]. In total, we identified 5942 and 2085 DEGs by comparing between *CmBBX22ox* and WT and between *CmBBX22-SRDX* and WT plants, respectively (Fig. 3B and C; Supplementary Data Table S3). To validate the expression of genes in transgenic and WT plants, a couple of genes were selected for qRT–PCR, including the drought-responsive genes *Cse_sc006945.1_g030.1* (*AT1G19640*, *JASMONIC ACID CARBOXYL METHYLTRANSFERASE*) and *Cse_sc000276.1_g070.1* (*AT3G47780*, *ATP-BINDING CASSETTE A7*) [28, 29]. qPCR and Integrative Genomics Viewer data showed that the gene expression patterns were consistent with the expression levels obtained based on RNA-seq analysis (Fig. 3D and E; Supplementary Data Fig. S3C and D).

To gain a functional insight into these DEGs, we performed Gene Ontology (GO) analysis, the results of which revealed an enrichment of GO terms in multiple biological processes and molecular functions, including water transmembrane activity, water channel activity, and response to abiotic stimulus, among genes differentially expressed between *CmBBX22ox* and WT (Fig. 3F), and responses to water deprivation, stress, and desiccation among those differentially expressed between *CmBBX22-SRDX* and WT (Fig. 3G). In addition, we also found that the chlorophyll binding, response to hormone, response to light, and cell death-associated pathways were enriched among the DEGs identified in both comparisons (Fig. 3F and G). Taken together, these results indicate that *CmBBX22* could influence pathways associated with stress and hormonal responses.

**Figure 4:**
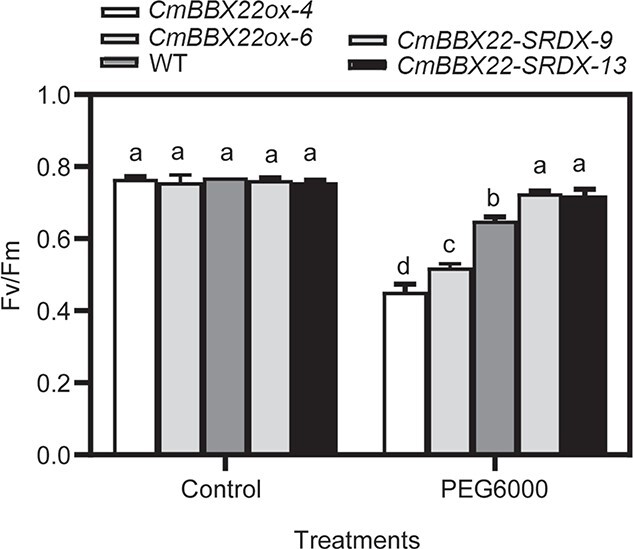
Chlorophyll fluorescence parameter *F*_v_*/F*_m_ in the basal leaves of 4-week-old *CmBBX22* transgenic plants and WT plants treated with water or 20% PEG6000 for 5 days. The values are shown as mean ± standard deviation from three independent experiments (*n* = 3). Different letters represent means that are significantly different according to Duncan’s test (*P* < 0.05).

Given that the chlorophyll binding pathway was found to be enriched with DEGs identified in both *CmBBX22ox* versus WT and *CmBBX22-SRDX* versus WT comparisons (Fig. 3F and G), and the chlorophyll fluorescence parameter *F*_v_*/F*_m_ gave insight into photosynthetic capacity [30] and is one of the most commonly used indexes for resistance assessment [31], we further examined *F*_v_*/F*_m_ in transgenic and WT plants. We were unable to detect any significant differences in *F*_v_*/F*_m_ ratios among *CmBBX22ox*, *CmBBX22-SRDX*, and WT lines (Fig. 4; Supplementary Data Fig. S4), indicating that CmBBX22 has no direct regulatory activity with respect to PSII activity in photosynthesis. In response to PEG6000 treatment, however, we did observe higher and lower amounts of *F*_v_*/F*_m_ in *CmBBX22-SRDX* and *CmBBX22ox* plants, respectively, compared with those in WT control plants (Fig. 4; Supplementary Data Fig. S4), consistent with the negative functions of *CmBBX22* in response to drought treatment.

### *CmBBX22*-mediated stomatal conductance is potentially involved in the negative regulation of chrysanthemum drought tolerance

**Figure 5:**
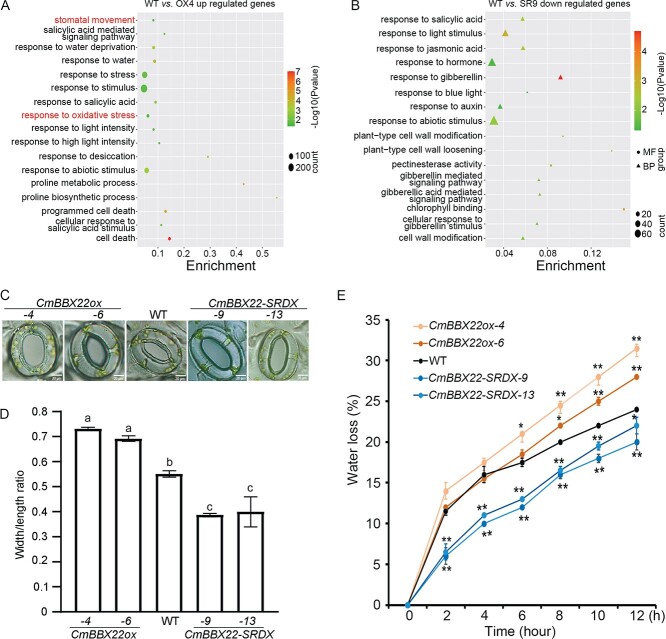
Stomatal conductance analysis. (A, B) Bubble plot showing the enrichment for GO pathways generated using 2501 upregulated genes in *CmBBX22ox* (A) and 998 downregulated genes in *CmBBX22-SRDX* (B). RNA-seq analyses were performed using *CmBBX22ox*, *CmBBX22-SRDX*, and WT plants cultivated under normal growth conditions. (C) Comparison of stomatal aperture between *CmBBX22* transgenic and WT plants under normal conditions. Representative images were taken under a microscope. Leaf peels were harvested from 35-day-old WT and transgenic plants grown on MS medium. Bar = 20 μm. (D) Ratios of stomatal aperture (width/length). Error bars represent means ± standard deviation of three biological replicates (*n* = 24). (E) Water loss from detached leaves of *CmBBX22* transgenic lines and WT plants. The water loss of detached leaves of 4-week-old plants was measured. Data are shown as means ± standard deviation of three replicates (*n* = 5). Asterisks represent significant differences compared with WT: ^*^P < 0.05, ^**^P < 0.01, Student’s test.

To further examine the function of *CmBBX22*-regulated genes, we performed GO analysis of those genes identified as being upregulated in *CmBBX22ox* plants and downregulated in *CmBBX22-SRDX* plants. In line with the findings obtained for GO enrichment of all DEGs (Fig. 3F and G), we observed the enrichment of GO terms associated with water, stress, light, and hormone responses and cell death (Fig. 5A and B). Previous studies have shown that potato plants in which the *Arabidopsis AtBBX21* gene is heterologously expressed are characterized by higher stomatal conductance associated with an increase in the size of the stomatal apertures [32]. Consistent with this observation, we similarly found that the stomatal movement pathway was enriched with genes that were upregulated in *CmBBX22ox* (Fig. 5A), thereby indicating that *CmBBX22* negatively regulates drought tolerance, potentially by modulating stomatal movement.

To further examine the putative influence of *CmBBX22* on stomatal movement, we examined stomatal conductance in *CmBBX22* transgenic and WT plants, and accordingly detected higher and lower stomatal conductance in *CmBBX22ox* and *CmBBX22-SRDX*, respectively, than in WT plants (Fig. 5C and D), thus indicating that *CmBBX22* plays a role in promoting stomatal opening. The findings of recent studies have also revealed that plants can optimize their CO_2_ uptake for photosynthesis and minimize water loss by altering the size of stomatal pore apertures [33], and thus we further investigated the rates of water loss in transgenic and WT plants. Observations revealed that *CmBBX22ox* and *CmBBX22-SRDX* plants show higher and lower rates of water loss, respectively, compared with those recorded in WT plants (Fig. 5E). In this regard, relative water content (RWC) is considered to be one of the most significant indices of dehydration tolerance [34], and hence we analyzed RWC in plants subjected to drought treatment. Whereas in untreated plants we detected no significant differences between transgenic and WT plants with respect to RWC, after 72 h of PEG6000 treatment the *CmBBX22-SRDX* and *CmBBX22ox* transgenic plants were found to have higher and lower RWCs, respectively, than WT plants (Supplementary Data Fig. S5).

Collectively, these observations thus indicate that *CmBBX22*-induced stomatal opening leads to high rates of water loss, thereby contributing to a reduction in the drought tolerance of chrysanthemum.

### The *CmBBX22*-mediated antioxidant response is potentially involved in the negative regulation of chrysanthemum drought tolerance

Exposure of plants to drought stress leads to excess production of toxic highly reactive oxygen species (ROS) that cause damage to plant cells. To scavenge ROS, plants deploy an efficient antioxidant defense system comprising non-enzymatic antioxidants and antioxidant enzymes such as superoxide dismutase (SOD), peroxidase (POD), ascorbate peroxidase (APX), and catalase (CAT) [35–37]. In the present study, we found that the response to oxidative stress pathway was enriched with genes identified as being upregulated in *CmBBX22ox* plants (Fig. 5A), thereby indicating that *CmBBX22* may play a role in regulating the response to oxidative stress.

**Figure 6:**
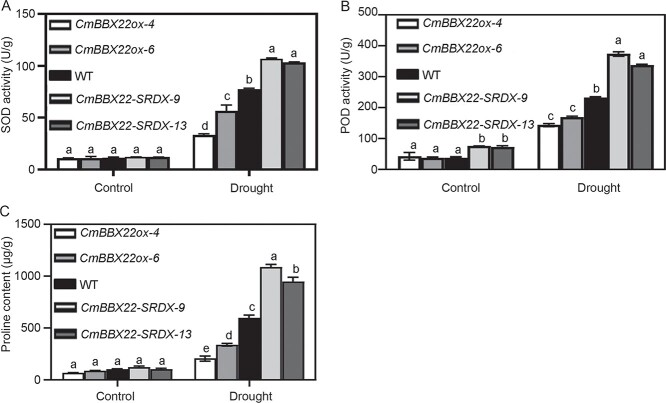
Determination of physiological indicators of transgenic and WT seedlings’ leaves after drought treatment. (A, B) Activity of leaf POD and SOD with/without drought treatment. (C) Contents of free proline in leaves. Values represent mean ± standard deviation (*n* = 3). Different letters represent significant differences between *CmBBX22* transgenic and WT plants (Duncan’s test, *P* < 0.05).

To assess the veracity of this supposition, we proceeded to examine SOD and POD enzyme activities, which revealed that whereas SOD activity was not affected by *CmBBX22* under normal conditions, POD activity was elevated in *CmBBX22-SRDX* plants (Fig. 6A and B). The activities of both SOD and POD were found to be altered in response to drought treatment, with higher and lower levels in *CmBBX22-SRDX* and *CmBBX22ox* plants, respectively, compared with the activities in WT plants (Fig. 6A and B). These observations would thus tend to indicate that *CmBBX22* negatively regulates drought tolerance by reducing ROS scavenging ability.

In plants, the accumulation of proline is a well-established metabolic response to drought and other stresses [38], which contributes to providing protection against different ROS [39]. On examining proline contents in transgenic and WT plants subjected to drought, we detected higher and lower levels of proline accumulated in *CmBBX22-SRDX* and *CmBBX22ox* plants, respectively, compared with those in WT plants, whereas no significant differences were detected under normal conditions (Fig. 6C). These findings indicate that *CmBBX22* may also contribute to the negative regulation of drought tolerance in chrysanthemum by modulating accumulation of the antioxidant proline.

## Discussion

Zinc finger-homeodomain genes comprise a relatively large family of transcription factors in plants (~15% of the total), with 32, 29, and 64 members having been identified in *Arabidopsis*, rice, and apple, respectively [22, 40, 41], and play a central role in plant growth and development and the response to environmental stimuli [8]. The BBX family is a subgroup of the zinc finger-homeodomain proteins, characterized by the presence of one or two N-terminal B-box domains [8]. Among these BBX proteins, it has been reported that members of the fourth subfamily play an important role in the responses to abiotic stress [13–15]. AtBBX22, which contains two N-terminal B-box domains, has previously been reported to positively regulate photomorphogenesis in *Arabidopsis* via its interaction with HY5 and COP1, and is degraded by COP1-mediated ubiquitination [21, 22]. Furthermore, heterologous expression of chrysanthemum *CmBBX22* in *Arabidopsis* has been found to promote delayed leaf senescence and enhance drought tolerance [20], while in this study we found *CmBBX22* to be a negative regulator of drought stress tolerance, mediated via regulation of stomatal conductance and antioxidant responses in chrysanthemum.

CmBBX22 is a transcription activator and transcriptional activation was mediated via the C-terminal region of this protein. In previous reports, the fragment between the B-box and CCT domains in BBX4, BBX8, and BBX16/COL7 had transcriptional activation activity in yeast cells [42–44], suggesting that an unidentified motif might reside within the fragment regions or C-terminal region. Recent research has found that the functional diversity within B-box proteins in structural group IV was caused by divergence of the C-terminal domains in *Arabidopsis* [45]. The motifs and residues in the C-terminal regions of BBX proteins need further research. Previous reports showed that AtARF5 is an activator with super-strong activities and is usually used as a positive control for the activity analysis of transcriptional activators [46, 47]. *35S::GAL4DB-AtARF5* protoplasts showed high LUC activity, indicating the success of our LUC assay. We found that the LUC activity in *Arabidopsis* mesophyll protoplasts transformed with *35S::GAL4DB-CmBBX22* was higher than in the negative control, indicating that CmBBX22 is a transcription activator. We also observed a lower LUC activity in *35S::GAL4DB-CmBBX22* protoplast than in the *35S::GAL4DB-AtARF5* positive control, suggesting that the transcription activity of CmBBX22 is lower than AtARF5, which is consistent with the reports that different transcriptional activators trigger gene expression at different levels [23, 48].

We established that overexpression of *CmBBX22* in chrysanthemum leads to a reduced tolerance to drought. Notably, this differs from the drought response phenotype obtained by heterologous expression of *CmBBX22* in *Arabidopsis*, the transformed plants of which showed enhanced drought tolerance [20]. Although seemingly counterintuitive, these observations are consistent with previously reported findings indicating that heterologous transformation of *Arabidopsis* with chrysanthemum genes sometimes leads to an opposite phenotype. For example, overexpression of *CmMYB2* in chrysanthemum results in earlier flowering, whereas heterologous expression of *CmMYB2* in *Arabidopsis* delays flowering [46, 49]. These observations would therefore tend to imply that chrysanthemum and *Arabidopsis* differ with respect to mechanisms underlying the regulation of plant development and stress responses. Unlike chrysanthemum, which is a perennial plant, *Arabidopsis* is an annual with a rapid life cycle and produces seeds within ~8 weeks. Consequently, it might be anticipated that chrysanthemums suffer higher levels of abiotic and biotic stresses during their life cycle. Thus, it can be speculated that, to adapt to adverse conditions, plants have evolved species-specific mechanisms that optimize plant growth to provide varying degrees of stress tolerance. However, further research is needed in this regard to elucidate the underlying mechanisms.

ROS play vital roles in the response to biotic and abiotic stimuli in plants. On encountering different stressful conditions, plants rapidly accumulate common ROS as a first layer of defense [50, 51]. However, when produced in excessive amounts, ROS can also cause irreversible cell damage. To counter these adverse effects, plants have evolved ROS scavenging mechanisms comprising two pathways involving non-enzymatic antioxidants and enzymatic components. Enzymatic components include SOD, CAT, APX, NADH, glutathione reductase (GR), guaiacol peroxidase (GPX), and monodehydroascorbate reductase, whereas glutathione (GSH), ascorbic acid, carotenoids, and osmolyte-proline are among the non-enzymatic antioxidants [35]. Constitutive expression of *OsGSTU4* (glutathione *S*-transferase) in *Arabidopsis* has been demonstrated to enhance the tolerance of transgenic lines to salinity and oxidative stresses by reducing the accumulation of ROS [52], whereas overexpression of a POD gene (*AtPER64*) in tobacco has been shown to reduce the root accumulation of aluminum and ROS [39]. In the present study, we found that the activities of both POD and SOD were significantly enhanced in *CmBBX22-SRDX* transgenic plants compared with WT plants (Fig. 6A and B), indicating that *CmBBX22* plays a role in the negative regulation of ROS scavenging, and further contributes to the negative regulation of drought tolerance. Proline is a osmoregulation substance, which will be accumulated largely under drought stress so as to reduce the damage to cells caused by drought and improve the drought tolerance of plants. We found that accumulation of proline in *CmBBX22-SRDX* plants was significantly higher than that detected in WT plants (Fig. 6C). These findings thus indicate that *CmBBX22* negatively regulates the antioxidant defense system and further diminishes the sensitivity of chrysanthemum to drought stress.

ROS regulate stomatal movements [53]. Besides ROS, it has been widely reported that many other factors could contribute to stomatal movement, including ABA, ethylene, nitric oxide (NO), and hydrogen sulfide (H_2_S) [54–56]. In addition, a recent report showed that high ROS levels could lead to ABA insensitivity, forming a feedback repression of continuously activated ABA signaling in guard cells [56]. ABA signaling plays a central role in the regulation of stomatal movements, particularly under water-deficit conditions. For example, *HAS1* is a negative regulator of ABA signaling and the *has1* mutant showed an ABA-hypersensitive stomatal closure phenotype [57]. The expression of *HAS1* was induced by *CmBBX22* in our transcriptome analysis, indicating possible roles of ABA signaling in *CmBBX22*-mediated stomatal opening. In conclusion, *CmBBX22*-mediated stomatal conductance may be controlled by integration effects of ROS, ABA signaling, and other factors. In the future, experiments related to functional verification of these factors in stomatal conductance are needed to further identify the underlying molecular mechanism of *CmBBX22*-mediated stomatal conductance.

In summary, we identified a *BBX* family gene, *CmBBX22*, in chrysanthemum and established that *CmBBX22* regulates drought stress responses in chrysanthemum. Transcriptome analysis and physiological measurements revealed that the *CmBBX22*-mediated ABA response, stomatal conductance, and antioxidant responses potentially contribute to the negative regulation of drought tolerance. Collectively, the findings of this study enable us to propose mechanisms whereby the transcriptional activator CmBBX22 negatively regulates drought tolerance in chrysanthemum.

## Materials and methods

### Plant growth conditions

The cut flower chrysanthemum cultivar ‘Jinba’ was obtained from the Chrysanthemum Germplasm Resource Conservation Center in Nanjing Agricultural University (Nanjing, China). Cuttings were planted in plug trays, and later the seedlings were transplanted in a 1:1 (v/v) mixture of vermiculite and peat in a greenhouse (day/night temperature 25/18°C, photoperiod 16 h, light intensity 120 μmol m^−2^ s^−1^, relative humidity 70%). We selected transgenic lines and WT plants that were at the same developmental stages for drought treatments. At first the plants were given adequate water and then water was withheld for 16 days. We next calculated the survival rate of the transgenic lines and the WT plants after 7 days’ recovery. For PEG6000-simulated drought treatment, transgenic and WT cuttings at the 8- to 10-leaf stage were treated with 20% w/v PEG6000, while those of control cuttings were treated with water [58, 59]. After treatment, plants were maintained in a greenhouse. Two leaves at the bottom of three seedlings were collected for further analysis.

### Subcellular localization analysis of CmBBX22

The *CmBBX22* ORF sequence was amplified using the primers covering *CmBBX22* open reading frame (ORF) regions and the construction of p35S::*GFP-CmBBX22* was performed as described previously [20]. For transient expression assays, *Agrobacterium* strain GV3101 carrying the constructs was infiltrated into 5- to 6-week-old *N. benthamiana* tobacco leaves. The tobacco plant was then cultured in darkness for 1 day followed by light treatment for 2 days. The green fluorescent protein (GFP) fluorescence signal was detected by laser confocal microscope (Zeiss, LSM800).

### Transcriptional activity analysis of CmBBX22

The *CmBBX22* ORF and amplicons were obtained using the Phusion High-Fidelity PCR Kit (New England Biolabs, Ipswich, MA, USA) using primer pairs containing EcoRI and BamHI sites. A set of pGBKT7-CmBBX22 fusions were generated by T4 DNA ligase (TaKaRa), including the pGBKT7-CmBBX22 construct (with full-length ORF sequence), pGBKT7-CmBBX22/Box1 construct, pGBKT7-CmBBX22/Box2 construct, pGBKT7-CmBBX22/Boxes (including CmBBX22/Box1 and CmBBX22/Box2), pGBKT7-CmBBX22/C construct, pCL1 (positive control), and pGBKT7 (negative control). The plasmids described above were transformed into Y2H Gold strain (Clontech) [60]. Transformants carrying either pGBKT7-CmBBX22, CmBBX22 residues or pGBKT7 were cultured on SD/−Trp medium, whereas the positive control pCL1 was cultured on SD/−Leu medium at 30°C. After 2–3 days, a single colony was selected and incubated on SD/−His−Ade medium with or without X-α-gal at 30°C. All primers used for this study are listed in Supplementary Data Table S1.

For the LUC assay, we recombined the pENTR™1A-CmBBX22 plasmid into the p35S::*GAL4DB* vector by LR recombination. The preparation and transformation of *Arabidopsis* protoplasts were performed as previously described [61]. Generally, 10 μg of p35S::*GAL4DB-AtARF5*, p35S::*GAL4DB*, or p35S::*GAL4DB-CmBBX22* plasmid was co-transformed with 5 × *GAL4-LUC* reporter gene into *Arabidopsis* protoplasts. After overnight incubation, the fluorescence values were obtained according to methods described previously [62].

### Transformation of chrysanthemum

The overexpression vector pMDC43-CmBBX22 and entry vector pENTR™1A-CmBBX22 were as described previously [20], and the CmBBX22 ORF from the last one was recombined into the pDEST_35S_SRDX_BCKH vector [63] via LR Clonase™ II enzyme mix (Invitrogen) to get the 35S::CmBBX22-SRDX construct as a chimeric repressor. For plant transformation, we introduced the 35S::CmBBX22 and 35S::CmBBX22-SRDX plasmids into *Agrobacterium* strain EHA105, and then generated transgenic chrysanthemum as described by Li *et al*. [64].

### Measurement of stomatal aperture

The leaves of WT and transgenic plants were harvested, and the epidermis close to the mid-vein was taken from the abaxial side. The epidermis was suspended on MES-KCl solution (10 mM MES, 50 mM KCl, pH 6.2) under lights for 2 hours at 22°C. Stomatal apertures of 24 guard cells in the epidermis of the abaxial surface were examined under a microscope, and three biological replicates were analyzed. The width and length of the stomatal aperture of guard cells per sample were measured using Olympus LAS V4.11 software. All measurements were performed between 8 a.m. and 12 noon, at a temperature of 22°C [65].

### Relative water content and water loss rate measurements

The transgenic chrysanthemum and WT seedlings at the 8- to 10-leaf stage were selected, and their roots were treated with 20% w/v PEG6000 solution. The third leaf (from the top) of three seedlings was collected at 0 h and 72 h of PEG treatment. The fresh weight of these leaves was measured. The leaves were then placed in deionized water for 24 hours at 22°C and re-weighed to give the turgid weight. Next, these leaves were baked at 65°C for 48 hours, then the dry weight was measured. RWC was calculated according to the method described previously [66]. Three biological replicates were performed.

The third fully expanded leaves from the top of *CmBBX22ox*, *CmBBX22-SRDX*, and WT plants were collected to estimate leaf water loss using the detached leaf method. Leaf fresh weight was measured immediately after detaching from the seedlings, then the leaves were stored at room temperature and weighed at 0, 2, 4, 6, 8, 10, and 12 hours after detaching. Experiments were performed with three biological replicates. The percentage of water loss was calculated as described by Ren *et al*. [67].

### Measurement of chlorophyll fluorescence parameters

All the expanded leaves at the bottom were collected. The chlorophyll fluorescence parameters were measured by pulse-amplitude modulation fluorometer (IMAGING-PAM, Walz, Germany) at room temperature (25°C), according to the method described by Su *et al*. [31]. The value of maximal photochemical efficiency (*F*_v_*/F*_m_) was calculated [31].

### Measurement of proline and antioxidant enzyme activity

SOD and POD activities and proline content were measured according to the manufacturer’s protocols (Nanjing Jiancheng Bioengineering Institute, Nanjing, China).

### RNA extraction and RNA-seq analysis

The fifth unfolded leaves of *CmBBX22-*overexpressing line *CmBBX22ox*-*4*, the *CmBBX22* chimeric repressor line *CmBBX22-SRDX*-9, and WT plants were collected at 30 days after transplanting. Total RNA was extracted with a Quick RNA Isolation Kit (Waryong, Beijing, China) and subjected to the Illumina sequencing platform at the Beijing Genomics Institute (Shenzhen, China) using an Illumina HiSeq™ 2000 instrument. Experiments were performed with three biological replicates. Adaptor sequences and low-quality reads were removed, and the clean data were then aligned to the *C. seticuspe* genome release CSE_r1.0 [25] using the kallisto [26] program. DEGs were identified using DEseq2 [27]. Genes showing *q* < 0.05 and |log2(fold change)| > 1 were considered to be differentially transcribed. Homologous genes of chrysanthemum in *Arabidopsis* were downloaded from the Mum Genome And Research Database ENtry (http://mum-garden.kazusa.or.jp/), and then GO analysis for these genes was performed using the online database agriGO [68]. Heat maps and Venn diagrams were generated using R (version 4.0.4).

### Quantitative real-time PCR validation

The primers used for validation were designed using Primer 5.0 software, and qRT–PCR was performed as previously described [69, 70]. The *EF1α* gene (GenBank AB548817.1) was selected as a reference [71]. Relative abundance was calculated using the 2^−ΔΔCt^ method [72]. The experiments were performed with three biological replicates, and each sample was examined with three technical replicates. All primers used in this study are listed in Supplementary Data Table S1.

### Abscisic acid treatment of detached chrysanthemum leaves

The third fully expanded leaves from the top of WT plants and the transgenic plants were detached and put into 2-mL EP tubes containing 2-mL water in the presence or absence of 50 μM ABA (Sigma–Aldrich, St Louis, MO, USA) for pretreatment for 12 hours in darkness, then transferred to empty 2-mL EP tubes as described previously [73]. Leaves were photographed at 0 and 6 hours respectively, and the water loss rates of the mock and ABA treatment groups were measured as described above. At least three replicated experiments were performed independently.

### Statistical analysis

Student’s *t*-test was used to determine significant differences in the results from *Arabidopsis* mesophyll protoplasts and water loss assays. Duncan’s multiple-range test was used for the analysis of significant differences in other results. All statistical analyses were performed with SPSS v19.0 (SPSS Inc., Chicago, IL, USA).

## Supplementary Material

supp_data_uhac181

## Data Availability

The data supporting this work are available in the paper and its supplementary information files. The data generated and analyzed in the study are available from the corresponding author upon request.
