# Supplementary material for: The BBX gene CmBBX22 negatively regulates drought stress tolerance in chrysanthemum
Source: Hortic Res. 2022 Aug 25;9:uhac181. doi: 10.1093/hr/uhac181 (PMC9630972; doi:10.1093/hr/uhac181)
Supplement: supp_data_uhac181 [file supp_data_uhac181.zip › Supplemental figures and tables.pdf]

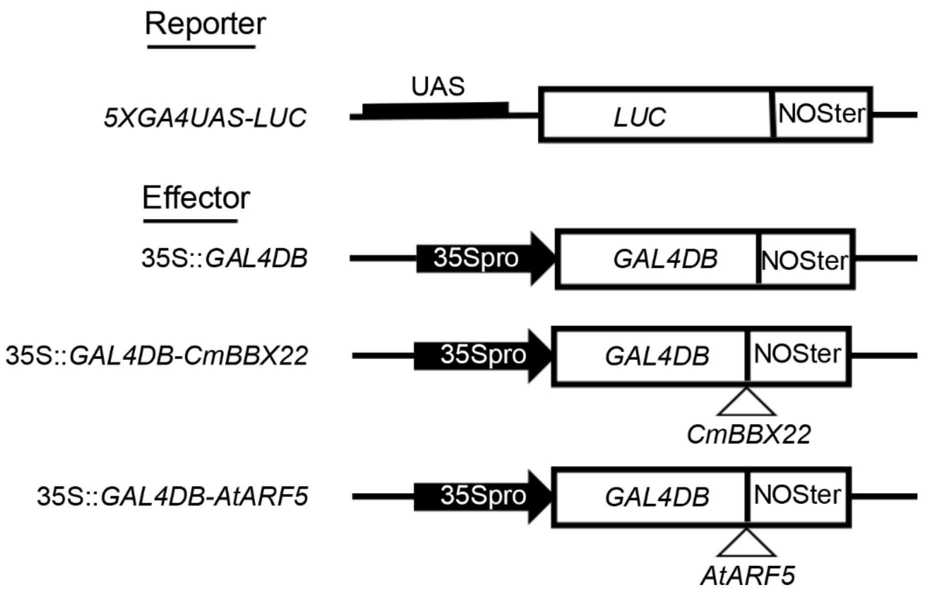

Fig. S1. Schematic diagrams of reporter and effectors used for luciferase assays.

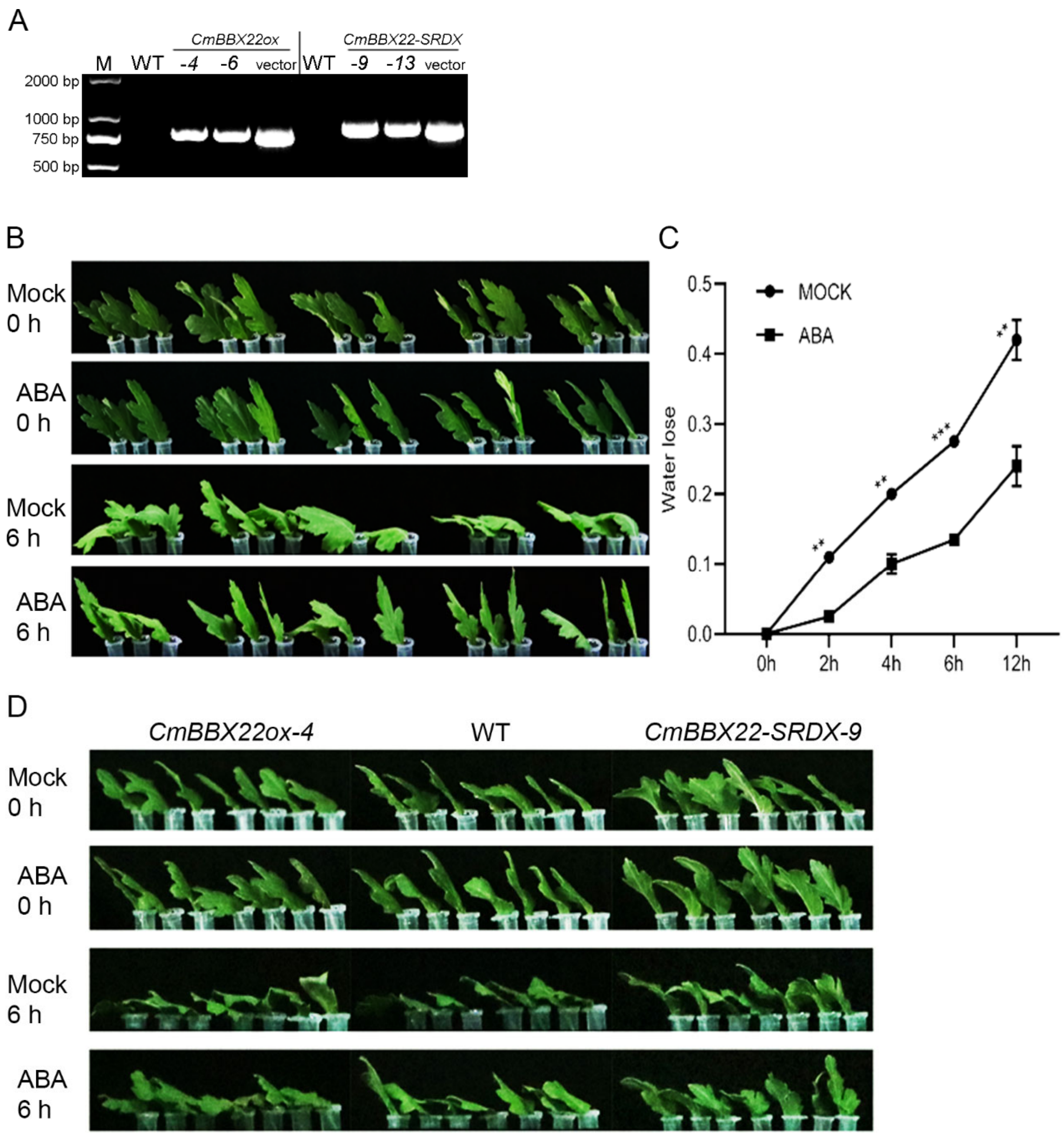

Fig. S2. The phenotype before and after ABA stress. (A)Agarose gel electrophoresis of PCR products on genomic DNA isolated from indicated plants. The vector used for plant transformation serves as positive control; WT plants used as negative control. (B and C)The phenotype (B) and statistic of water lose in leaves (C) of wild type plants in the pre-experiment with and without ABA treatment. (n = 15), error bars indicate standard deviation (SD), Significant differences were determined by students' test, ** $P < 0.01$, ***$P < 0.001$. (D) The phenotype of transgenic plants and wild type plants at 0h and 6h before and after ABA treatment (n=7).

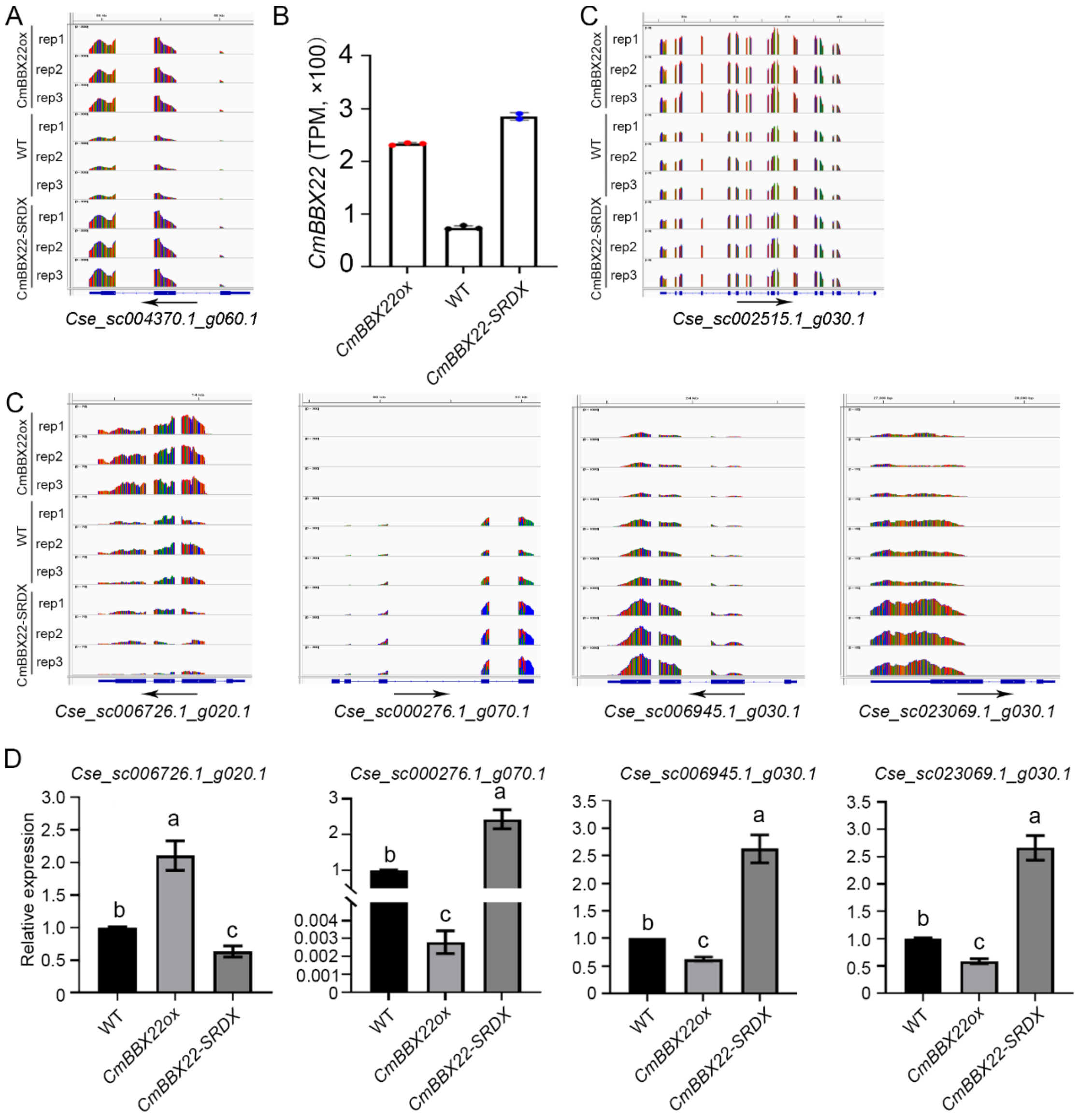

Fig. S3. (A) Genome browser traces of RNA-seq results of *CmBBX22* in *CmBBX22ox*, *CmBBX22-SRDX* and WT plants. (B) The expression values of *CmBBX22* attained from RNA-seq analysis. (C) Genome browser traces of the reference gene *EF1α* (Cse_sc002515.1_g030.1) and RNA-seq results of differentially expressed genes in *CmBBX22ox*, *CmBBX22-SRDX* and WT plants. (D) qRT-PCR assay to examine the expression of genes in *CmBBX22ox*, *CmBBX22-SRDX* and WT plants. The chrysanthemum *EF1α* was used as the reference gene for normalization. Error bars indicated the S.D. (n = 3). Different letters indicate statistical difference ($P < 0.05$).

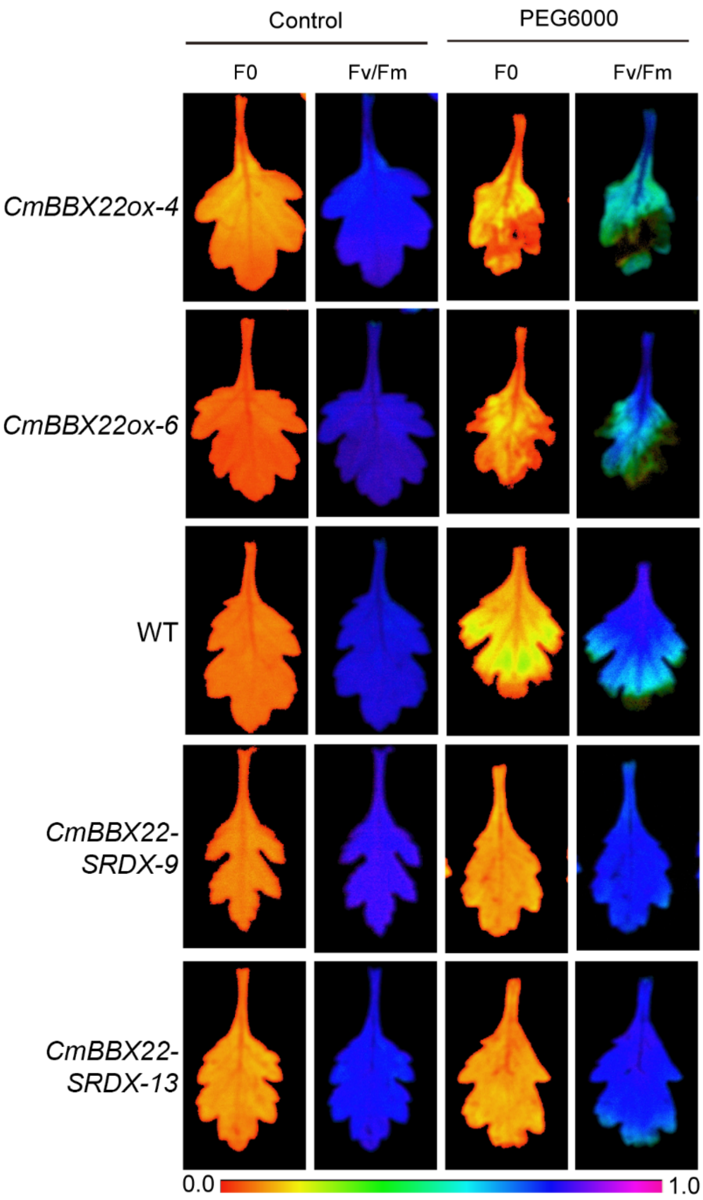

Fig. S4. Images of *F0* (left panel) and *Fv/Fm* (right panel) of leaves from the *CmBBX22* transgenic plants and WT plants under water (control) and 20% PE6000 treatment. The pseudocolored bar depicted at the bottom of the image ranges from 0 (Red) to 1 (purple).

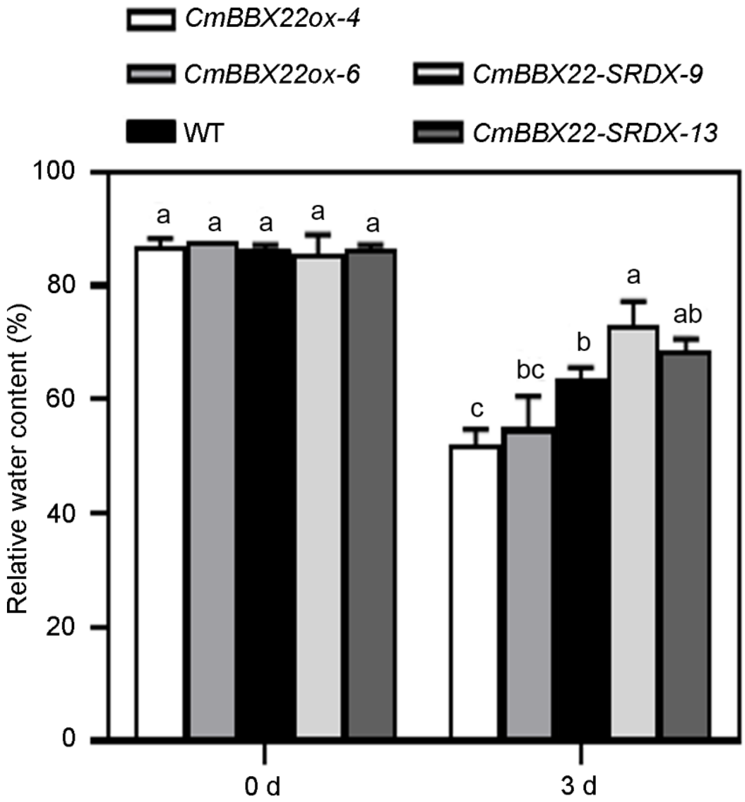

Fig. S5. Relative water content of *CmBBX22* transgenic and WT plants with or without 3-day PEG6000 treatment. The values were shown as means ± SD from two independent experiments (n = 8). Different letters represent significant differences between of *CmBBX22* transgenic and WT plants (Duncan's test, $p < 0.05$).

Table S1, primers used for qRT-PCR and vector construction.

Primer name	Primer sequence	
Cse_sc072230.1_g020.1-F	GCTGTGGGTTAAGTGGGTTC	qRT-PCR
Cse_sc072230.1_g020.1-R	TTCCAACTCCATGCTCCATGT	qRT-PCR
Cse_sc013082.1_g010.1-F	TCGCCGTCGAAATCAAACAA	qRT-PCR
Cse_sc013082.1_g010.1-R	GCCGAGATATCCGGTGATGA	qRT-PCR
Cse_sc000276.1_g070.1-F	CGGCAGCATTCAAACATCCC	qRT-PCR
Cse_sc000276.1_g070.1-R	TGGATGGCTGCTGTAAAAGGT	qRT-PCR
Cse_sc006945.1_g030.1-F	TTGTCCCCTAACCCATGTGC	qRT-PCR
Cse_sc006945.1_g030.1-R	TGGTGCATAATAAGGGGCGT	qRT-PCR
Cse_sc023069.1_g030.1-F	CTCAAAGAGGTACGAGGCGA	qRT-PCR
Cse_sc023069.1_g030.1-R	TGCACTCGTTGCGTCATTTT	qRT-PCR
Cse_sc010657.1_g010.1-F	AAAGATGGAGCTACGGGGTC	qRT-PCR
Cse_sc010657.1_g010.1-R	GCGACACACCTTGAGGAGAA	qRT-PCR
Cse_sc006726.1_g020.1-F	CGTTGATAGCATCACCGCTA	qRT-PCR
Cse_sc006726.1_g020.1-R	TGGCAGTGTTTTCCCCATGA	qRT-PCR
Cse_sc002677.1_g030.1-F	TGTATGGCATGTTTGAGCCCT	qRT-PCR
Cse_sc002677.1_g030.1-R	ATCAGCGGTTGAGAACTCGT	qRT-PCR
EF1a-F	TTTTGGTATCTGGTCCTGGAG	qRT-PCR
EF1a-R	CCATTCAAGCGACAGACTCA	qRT-PCR
B22-BD-F	CCGGAATTCATGAAGATTCAGTGCAACG	Y2H
AD(BD)B22-BOX1-R	GCGGATCCCGGCATCAGAGTTGG	Y2H
AD(BD)B22-BOX2-F	CGGAATTCAAGATGCCTAAGTGTG	Y2H
AD(BD)B22-BOX2-R	GCGGATCCCTACTTTGACTCCAG	Y2H
AD(BD)B22-C-F	CGGAATTCGGGGCTGAAGCTGC	Y2H
B22-BD-R	CGCGGATCCCTTGCCTTCGTCTTTTTAGGC	Y2H

Table S2, summary of reads and differentially expressed genes (DEGs) in different samples.

Samples	Number of total clean reads	Number of reads pseudoaligned	Mapped ratio	Numbers of DEGs (*vs.* WT)
CmBBX22ox rep1	21,236,397	13,964,624	0.66	
CmBBX22ox rep2	21,177,027	13,831,415	0.65	5942
CmBBX22ox rep3	21,310,539	13,875,626	0.65	
CmBBX22-SRDX rep1	21,343,926	12,237,353	0.57	
CmBBX22-SRDX rep2	21,051,364	13,981,879	0.66	2085
CmBBX22-SRDX rep3	21,068,723	14,149,123	0.67	
WT rep1	21,177,345	14,382,609	0.68	
WT rep2	21,217,133	14,400,112	0.68	
WT rep3	21,193,229	14,424,263	0.68	
